# Timed Pulses in DNA Strand Displacement Reactions

**DOI:** 10.1021/jacs.3c06664

**Published:** 2023-09-15

**Authors:** Juliette Bucci, Patrick Irmisch, Erica Del Grosso, Ralf Seidel, Francesco Ricci

**Affiliations:** † Department of Chemical Sciences and Technologies, University of Rome, Tor Vergata, Via della Ricerca Scientifica, 00133 Rome, Italy; ‡ Molecular Biophysics Group, Peter Debye Institute for Soft Matter Physics, Universität Leipzig, 04103 Leipzig, Germany

## Abstract

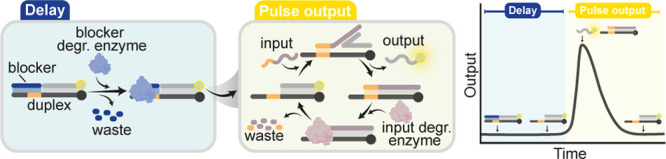

Inspired by naturally occurring regulatory mechanisms that allow complex temporal pulse features with programmable delays, we demonstrate here a strategy to achieve temporally programmed pulse output signals in DNA-based strand displacement reactions (SDRs). To achieve this, we rationally designed input strands that, once bound to their target duplex, can be gradually degraded, resulting in a pulse output signal. We also designed blocker strands that suppress strand displacement and determine the time at which the pulse reaction is generated. We show that by controlling the degradation rate of blocker and input strands, we can finely control the delayed pulse output over a range of 10 h. We also prove that it is possible to orthogonally delay two different pulse reactions in the same solution by taking advantage of the specificity of the degradation reactions for the input and blocker strands. Finally, we show here two possible applications of such delayed pulse SDRs: the time-programmed pulse decoration of DNA nanostructures and the sequentially appearing and self-erasing formation of DNA-based patterns.

## Introduction

Many biological processes exhibit a variety of elegant mechanisms that allow their flexible control over time in response to varying environmental conditions. Finely tuned temporal programs of gene expression^1,2^ and mRNA levels, for example, result from the combination of complex regulatory mechanisms, such as feedback loops,^3,4^ delays,^5−8^ and pulse-generating systems.^9^ Such temporal patterns and pulse changes can usually occur over a very wide range of time scales (from minutes to days) and can dynamically change in response of different molecular inputs.^10,11^ Thanks to the nonequilibrium nature of biological systems, communication between living cells is also often achieved through the transient production of signaling molecules with specific temporal features and with different widths and time periods (or delays).^12,13^ Ultimately, these temporally encoded mechanisms enable the efficiency, order, and compartmentalization of gene expression, cellular signaling, differentiation, and communication, and form the basis for the ability of cells and living organisms to adapt to various external perturbations and stimuli.^14,15^

Recently, a number of synthetic man-made systems have been described that recapitulate in vitro some of the above mechanisms as a way to temporally control chemical systems, polymers, hydrogels, and self-assembly reactions.^16−20^ Among these examples, the programmability of DNA–DNA interactions with their predictable base pairing has been shown to be particularly useful for rationally designing DNA-based reactions, devices, and tools that can be temporally programmed.^21−25^

Examples in this direction include the design of DNA-based timed logic circuits that can achieve sequential release of DNA sequences with adjustable delays, store temporal information about molecular signals or induce the temporal assembly of DNA structures.^26−30^ ATP-dependent enzymatic reactions have also been used to control the time at which DNA-based polymers and structures can form.^31−34^ Furthermore, we have recently demonstrated the possibility of using enzymatic reactions to achieve pulse-like outputs in DNA-based strand displacement reactions (SDRs)^35^ and, more recently, nucleic acid blockers to temporally program the onset of SDRs.^36^

**Figure 1:**
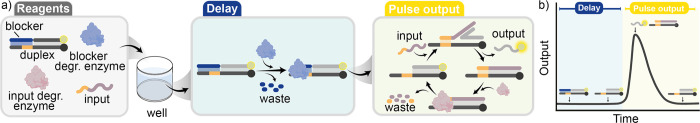
Programmable timed pulses in DNA strand displacement reactions. (a) Initial configuration of the system includes a target duplex, an input strand capable of displacing the output strand from the target duplex, and a blocker strand (blue) that is complementary to the toehold domain of the DNA duplex and can effectively inhibit the SDR. The reaction starts with the addition of an enzyme that specifically degrades the blocker strand. Once the blocker is completely degraded, the input strand triggers the SDR. Subsequently, the input strand itself is enzymatically degraded, allowing the system to restore the original state. (b) By tuning the blocker and the input strand degradation rate, the delay and the pulse output can be programmed.

Despite the above examples, the possibility of programming DNA-based systems that exhibit temporal pulse features with programmable delays, such as naturally occurring genetic circuits and signaling pathways, has not yet been demonstrated. A similar mechanism would ultimately be useful to create temporally encoded synthetic devices and structures. Motivated by the above consideration, we demonstrate here a strategy to achieve temporally programmed pulse output signals in DNA-based SDRs. To achieve this, we used an input strand that, once bound to its target duplex, can be gradually degraded by an enzyme, resulting in a pulse output signal. We then designed a blocker strand that can efficiently prevent strand displacement and determine the delay with which the pulse reaction can be generated (Figure 1)

## Results and Discussion

To achieve timed pulses in DNA SDR, we first used RNA blocker strands, RNA input strands, and the endoribonuclease RNase H as the corresponding degradation enzyme. RNase H is an enzyme capable of degrading RNA strands only when these are bound within DNA–RNA heteroduplexes.^37^ We designed the RNA blocker strand to bind to a 20-nt single strand of the original DNA duplex so that it efficiently prevents the binding of the input strand to the toehold domain and thus the triggering of the SDR. The delayed pulse SDR is activated by the addition of RNase H, which degrades the RNA blocker over time and induces the SDR with a programmable delay that depends on the rate of degradation of the blocker. Once the RNA input strand displaces the output strand and binds to the target duplex, it is also degraded over time, resulting in a pulse output signal (i.e., the target duplex returns to its original state).

**Figure 2:**
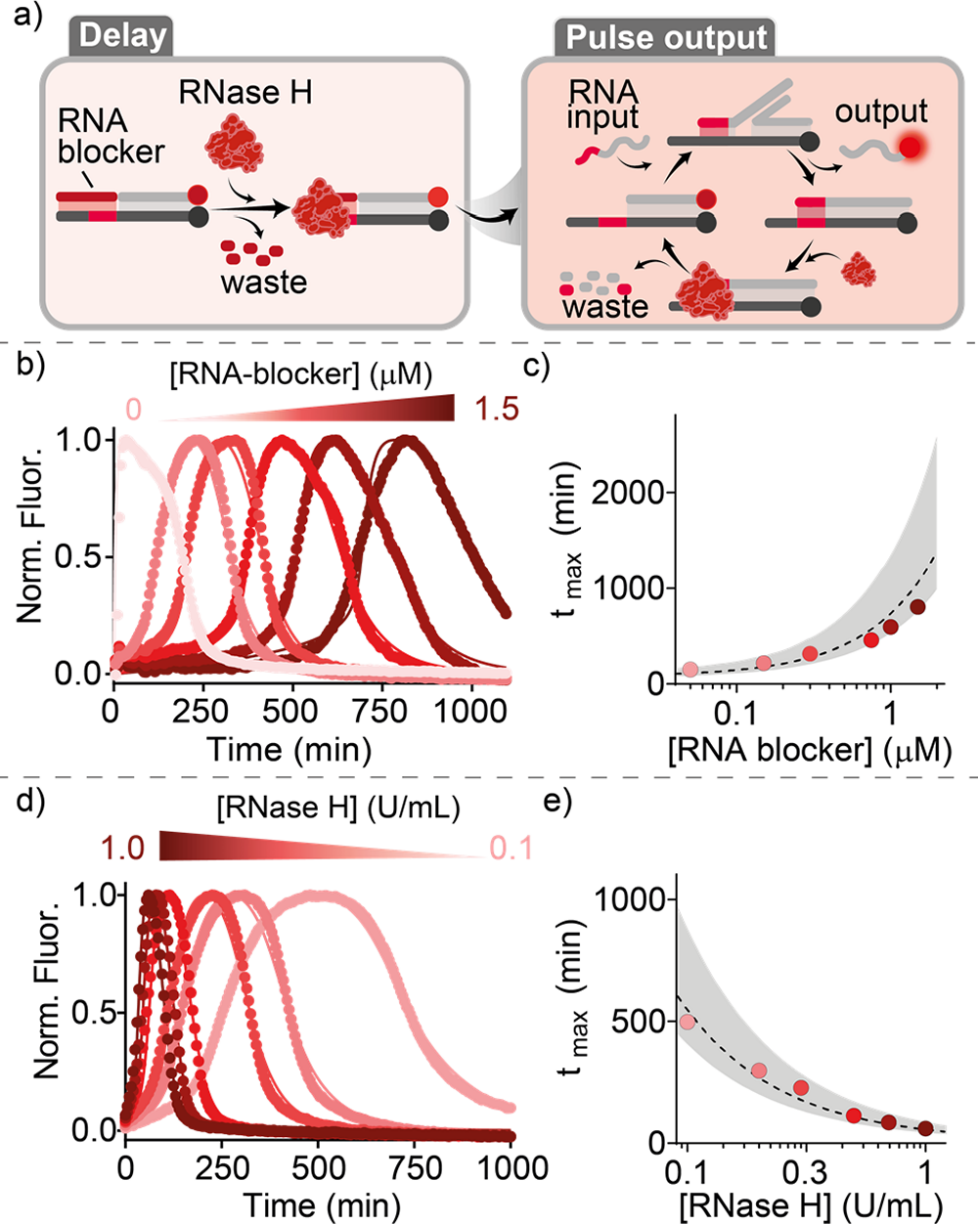
Timed pulses in SDRs using RNase H. (a) Scheme of the reaction steps. (b) Pulse SDRs at different concentrations of RNA blocker from 0 to 1.5 μM. (c) *t*_max_ values (defined as the time required to reach the maximum signal of SDR) vs RNA blocker concentration. (d) Pulse SDRs at different concentrations of RNase H (from 0.1 to 1.0 U/mL). (e) *t*_max_ values vs RNase H concentration. In the graphs, dots represent experimental values while solid/dashed lines represent fits (b, d) and predictions (c, e) obtained with the kinetic model (see SI, Section 2c). The shaded areas (c, e) represent the 95% confidence interval. The experiments were performed in a solution of Tris-HCl 20 mM, MgCl_2_ 10 mM, EDTA 1 mM, and pH 8.0, containing the target duplex (50 nM) and the input strand (150 nM) at *T* = 30 °C. Unless otherwise noted, blocker strand and RNase H were used at 150 nM and 0.3 U/mL, respectively.

To monitor SDR in real time, we labeled the target duplex with a fluorophore and a quencher so that the displaced output would result in an increase in the fluorescence signal (Figures 2a and S1). Initially, we performed SDRs using different concentrations of the RNA blocker strand from 0 (in absence of RNA blocker) to 1.5 μM, keeping all the other strands (target duplex and input strand) at a fixed concentration. In this way, we were able to modulate the *t*_max_ values (defined here as the time required to reach the maximum SDR signal) from 30 ± 5 to 805 ± 78 min (Figure 2b,c). Similarly, we were able to efficiently modulate *t*_max_ values by varying the concentration of RNase H (keeping the blocker strand at a fixed concentration) (Figure 2d,e). In addition to being able to precisely control the *t*_max_ value, we can also modulate the width of the pulse. In particular we demonstrate that at a fixed concentration of RNase H and blocker strand, we can increase the pulse width from 261 ± 36 to 1110 ± 84 min by increasing the concentration of the RNA input from 0.3 to 1.0 μM, respectively (Figure S2).

To better understand the achieved timed pulse SDR, we developed a model based on a simplified reaction scheme to describe the obtained kinetics. In this scheme (Figures S3 and S4), individual reaction steps were considered irreversible, and the enzyme was assumed to operate under saturation conditions. In particular, the model accounts for RNA blocker and RNA input degradation, toehold-mediated strand displacement, as well as hybridization of free single strands (for further information see Supporting Information Section 2c, and Figures S5 and S6). A global fit of the model to the experimental data showed excellent agreement for all of the blocker and enzyme concentrations tested (Figure 2b,d, solid lines). Moreover, the fit provided the rate constants within the simplified reaction scheme, which allows predicting the time required to achieve the maximum signal given the experimental conditions (Figure 2c,e, dashed lines). Overall, the good agreement between the model and experimental data demonstrates that the established pulse-DNA SDR follows the anticipated pathway. Furthermore, our modeling suggests that enzyme inhibition is negligible under the employed experimental conditions, as the obtained enzyme activity rate constants (see Figure S6) do not exhibit a clear trend, except for minor deviations observed at high RNA blocker concentrations. Nevertheless, it is worth noting that at higher blocker concentrations significant enzyme inhibition can be expected due to the accumulation of waste products. Moreover, we emphasize that leak reaction pathways, where the input strand initiates SDRs before entire blocker strand degradation, play only a minor role. This conclusion is supported by the observation of only a slight increase in the intensity of the signal before the pulse signal initiates.

**Figure 3:**
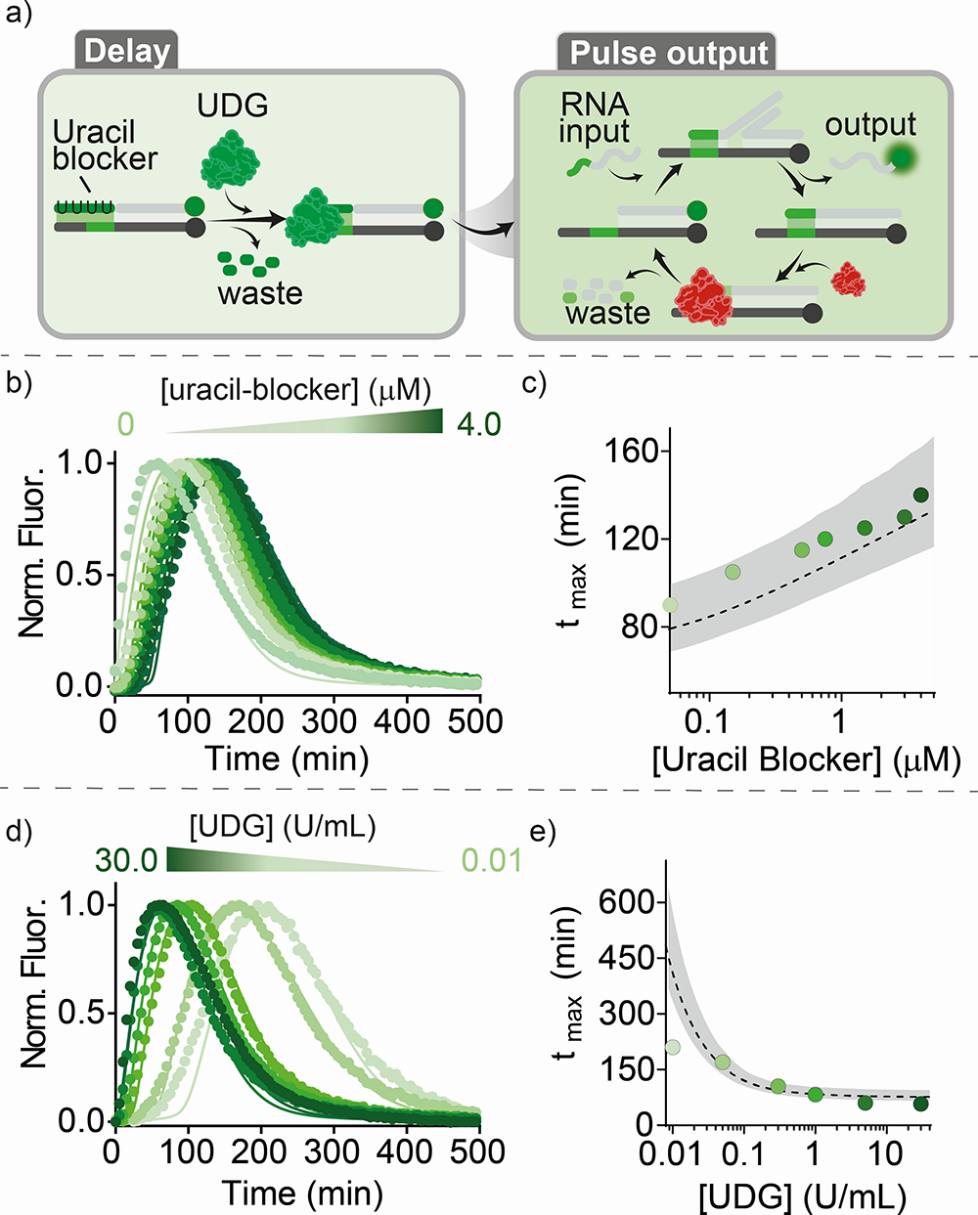
Timed pulses in SDR using UDG and RNase H. (a) Scheme of the reaction steps. (b) Pulse SDRs at different concentrations of uracil blocker from 0 to 4 μM. (c) *t*_max_ values vs uracil blocker concentration. (d) Pulse SDRs at different concentrations of UDG (from 30 to 0.01 U/mL). (e) *t*_max_ values vs UDG concentration. In the graphs, dots represent experimental values, while solid/dashed lines represent fits (b, d) and predictions (c, e) obtained with the kinetic model (see SI, Section 2d). The shaded areas (c, e) represent the 95% confidence interval. The experiments were performed in a solution of Tris-HCl 20 mM, MgCl_2_ 10 mM, EDTA 1 mM, and pH 8.0, containing the target duplex (50 nM) and the input strand (150 nM) at *T* = 30 °C. Unless otherwise noted, blocker strand was used at 150 nM and both UDG and RNase H were used at 0.5 U/mL.

To enable programmable coordination of multiple reaction events, as occurs in complex gene regulatory cycles, we designed an orthogonal system that used a different enzyme to degrade the blocker (Figures 3a and S7). To this end, we used the enzyme uracil DNA glycosylase (UDG), a repair enzyme that catalyzes the excision of uracil bases in DNA strands creating abasic sites.^38^ As the blocker (uracil blocker, green, Figure 3a), we designed a DNA strand containing four deoxyuridine mutations. After UDG activity, the four produced abasic sites destabilize the complex between the blocker strand and the target duplex causing the dehybridization of the blocker and triggering the downstream pulse SDR. As with the previous system, we were able to obtain pulse outputs with programmable delays varying either the concentration of the blocker strand (Figure 3b,c) or that of UDG enzyme (Figure 3d,e). The lower efficiency of the programmed pulse delay is likely due to the different mode of action of UDG that, contrarily to RNase H, degrades both the free uracil blocker and the bound one, resulting in an overall faster blocker degradation rate.

To understand the obtained kinetics for this system, we developed an extended model that accounts for the different mechanics of the uracil blocker degradation by UDG (Supporting Information, Section 2d and Figures S8 and S9). Similar to the RNA blocker-based system, the model reproduced the experimental data for all of the tested blocker and enzyme concentrations (Figure 3b,d, solid lines) and enabled prediction of the observed time delays (*t*_max_) (Figure 3c,e, dashed lines).

**Figure 4:**
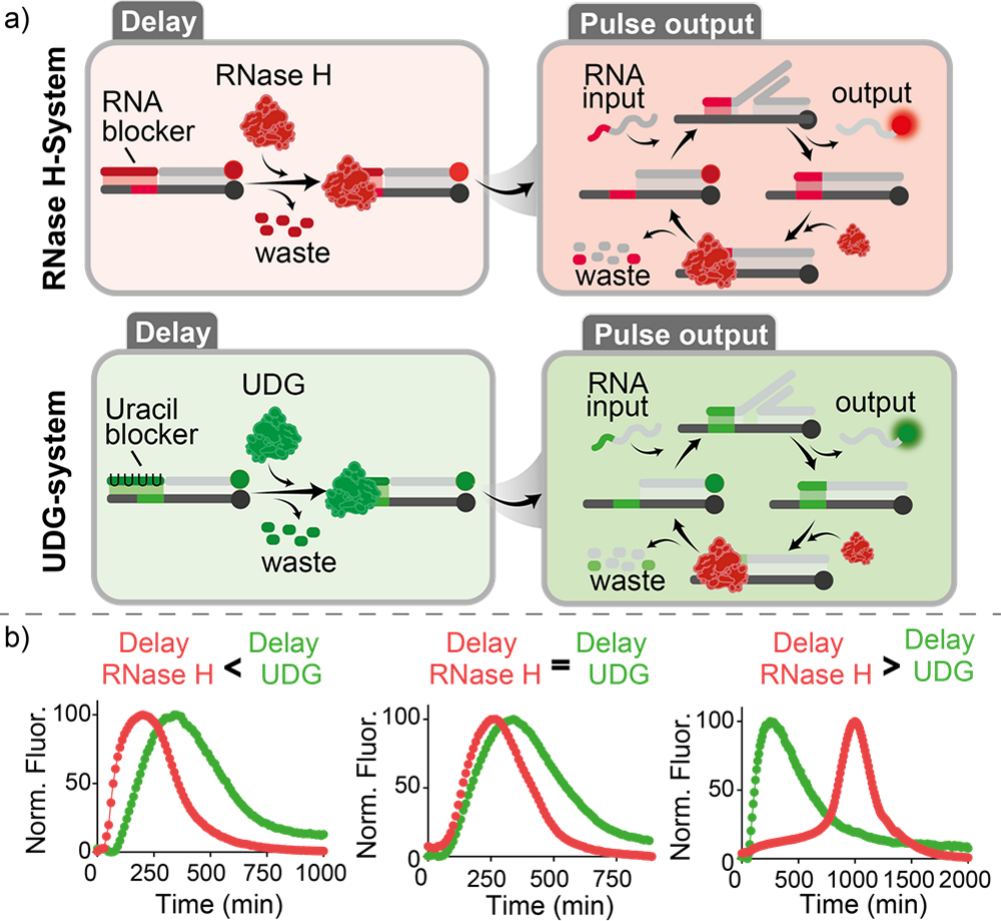
Orthogonal timed pulse SDRs. (a) Schemes of the two orthogonal timed pulse SDRs. (b) Examples of the timed pulse SDRs activated independently at different times by using different concentrations of blocker strands. The experiments were performed in a solution of Tris-HCl 20 mM, MgCl_2_ 10 mM, EDTA 1 mM, and pH 8.0, containing both the target duplexes (50 nM) and the input strands (150 nM) at *T* = 30 °C. The following concentrations of blocker strands are employed: left: [RNA blocker] = 0.05 μM, [UDG blocker] = 0.4 μM; middle: [RNA blocker] = 0.15 μM, [UDG blocker] = 0.15 μM; and right: [RNA blocker] = 1.0 μM, [UDG blocker] = 0.15 μM. RNase H was fixed for all experiments at 0.5 U/mL. [UDG]: left: 0.5 U/mL; middle 0.01 U/mL; and right: 0.3 U/mL).

The two systems described above are fully orthogonal, that is, each is labeled with a different fluorophore and uses different input strands and blocker-enzyme pairs (Figure S10). To demonstrate that the two systems presented above can be operated orthogonally to achieve pulse-delayed outputs from two SDRs in the same solution, we performed time-course fluorescence experiments with both systems in the same solution using different blocker concentrations (Figure 4a). In this way, the order of onset of the various pulse outputs can be modulated independently in a programmable manner (Figure 4b).

**Figure 5:**
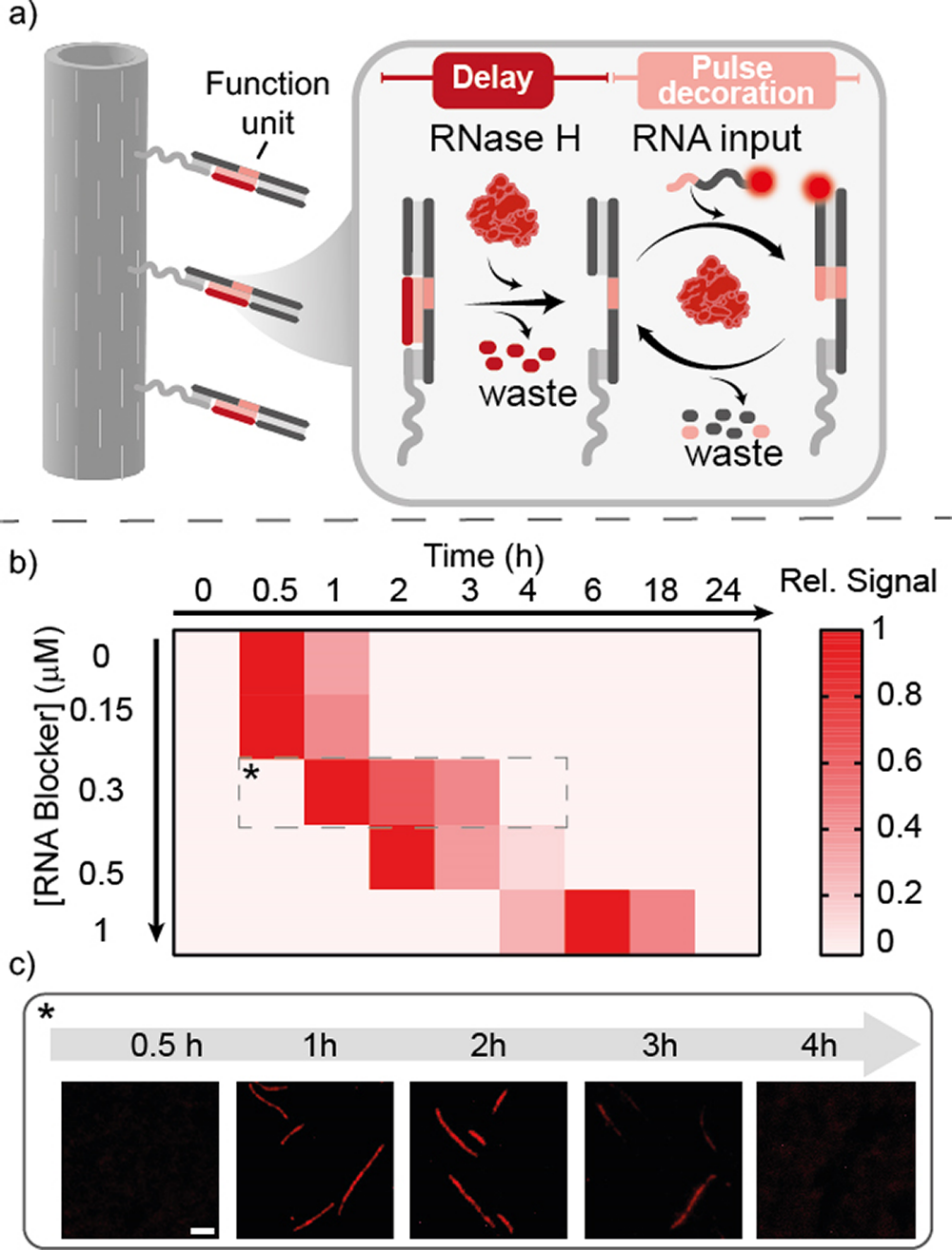
Timed pulse decoration of DNA nanostructures. (a) Scheme of the reactions on the DNA structure. The DNA tile displays a 20-nt ssDNA overhang that serves as anchor for the target duplex. An RNA blocker strand and an RNA input strand labeled with a fluorophore tag (Cy3) were used together with RNase H as the degradation enzyme. (b) Analysis of the pixel intensity of the structures at different concentrations of the RNA blocker strand. (c) Fluorescence microscopy images of the DNA structures at different times after RNase H addition using a RNA blocker strand concentration of 0.3 μΜ. Scale bar 2 μm. The experiments shown in this figure were performed in a solution of Tris-HCl 20 mM, MgCl_2_ 10 mM, EDTA 1 mM, and pH 8.0 at *T* = 30 °C, using 100 nM of assembled structures, 50 nM of target duplex, 150 nM input strand, 3 U/mL RNase H, and the indicated concentration of the RNA blocker strand.

To demonstrate the applicability of the proposed strategy for achieving a timed pulse SDR in more complex reaction systems, we developed two possible applications. First, we employed our pulse SDR to achieve the timed pulse decoration of DNA nanostructures. To this end, we assembled nonfluorescent tubular DNA nanostructures using a tile-based approach. Specifically, we used DNA tiles formed through the hybridization of five different DNA strands with four sticky ends, which enabled their self-assembly into tubular structures.^39,40^ We also re-engineered one of the tile-forming strands to have an additional 20-nt ssDNA overhang that serves as an anchor for binding a duplex with a toehold portion (Figure 5a). To achieve timed pulse decoration of these structures, we added to the solution containing the tubular structures an RNA blocker strand that can hybridize to the toehold domain of the target duplex and a fluorophore-labeled RNA input strand (Cy3). The addition of RNase H initiates the timed pulse decoration of the structures. Specifically, RNase H causes the degradation of the RNA blocker over time, allowing the Cy3-conjugated RNA input strand to bind to the target duplex on the structure at a specific time point (Figure 5a). Once the RNA input strand is also degraded, the original duplex is restored and the structures return to their undecorated state (Figure 5a). By changing the concentration of both the RNA blocker strand and the RNase H, we were able to control the timing of pulsed decoration over a 24 h period (Figures 5b,c, S11, and S12). To further prove the versatility of our strategy, we achieved the timed pulse decoration of DNA nanostructures using UDG as a degrading enzyme for the blocker strand. As with the previous system, we obtained programmable control over the timing of pulsed decoration by varying the UDG concentration (Figure S13).

**Figure 6:**
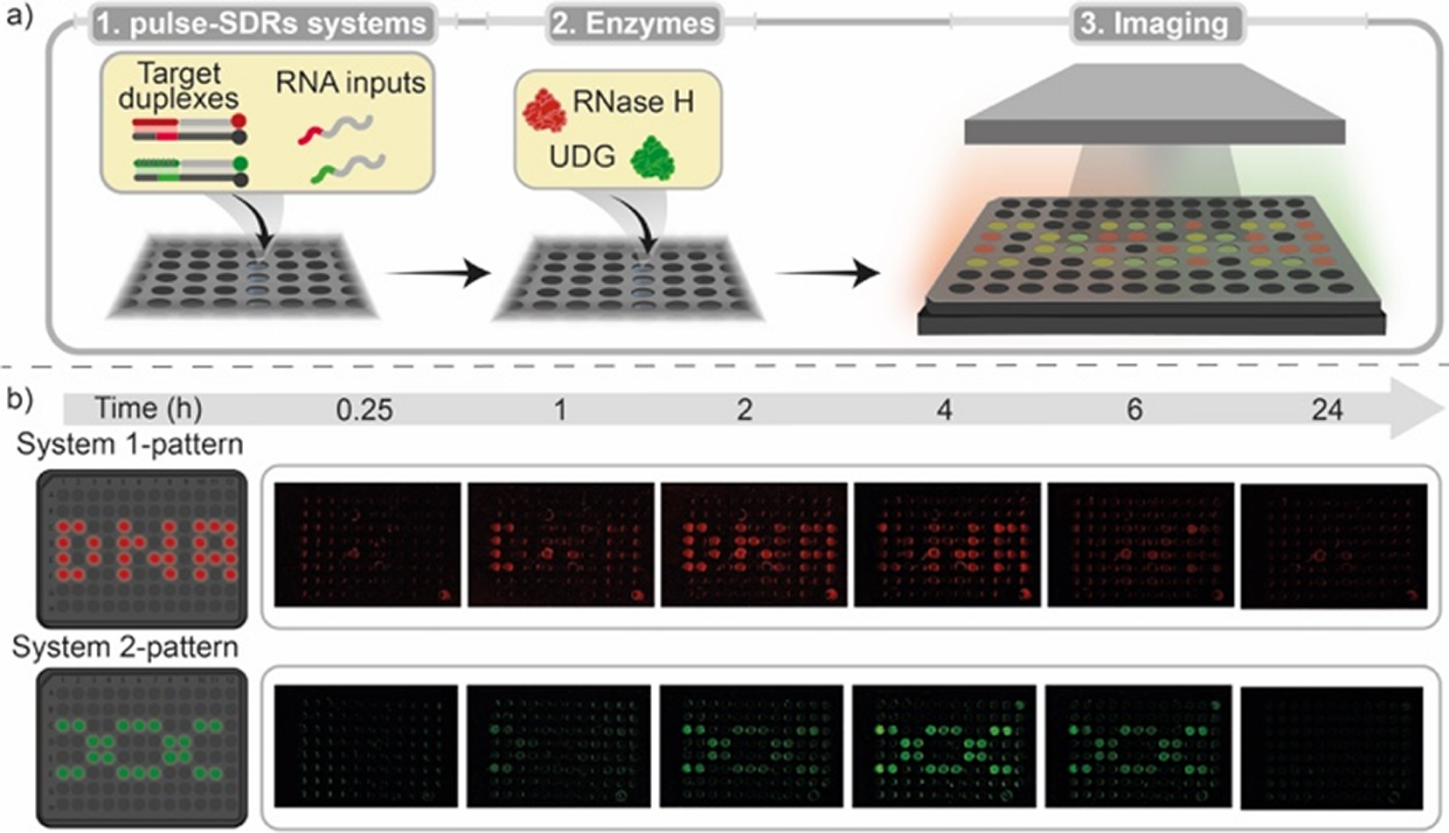
Sequentially appearing and self-erasing DNA patterns. (a) Scheme of the SDR systems used to achieve sequentially appearing and self-erasing patterns in a 96-well plate. (b) Sequential images of the 96-well plate at different times resulting in the programmed self-erasing patterns. The experiments shown in this figure were performed in a solution of Tris-HCl 20 mM, MgCl_2_ 10 mM, EDTA 1 mM, and pH 8.0 at *T* = 30 °C. Each well contains a fixed concentration of target duplexes (50 nM) and RNA inputs (150 nM) for both systems and different concentrations of the two blocker strands. The two degradation enzymes (RNase H and UDG) were added at the same time in all of the wells to achieve the sequential appearance and self-erasing pattern.

As a second application, we demonstrate the design of a sequentially appearing and self-erasing pattern. To do that, we used the two orthogonal pulse-SDRs characterized before (Figures 2 and 3). We added in each well of a 96-well plate a fixed concentration of the target duplexes prehybridized with different concentrations of their respective blocker strands and RNA input strands. The concomitant addition of a fixed concentration of RNase H and UDG in desired wells initiates the reactions, allowing to display the sequential and self-erasing graphical patterns “DNA” (RNase H-based system, red) and a stylized helix (UDG and RNase H-based system, green). Using an imaging system for the visual readout and a plate reader for confirmation of the results, we were able to observe the sequential appearing of the programmed patterns over a 4 h time period and their self-erasing after 6 h (for RNase H system) and 24 h (for UDG system) (Figures 6a and S14, Supporting Information Video 1). Of note, system #1 (pattern “DNA”, red) was programmed to appear and self-erase before system #2 (stylized helix, green).

## Conclusions

In summary, we have described here an enzyme-driven strategy for achieving time-programmed pulse output signals in DNA SDRs. Specifically, we have developed two orthogonal pulse SDRs, each triggered by a specific enzyme (RNase H and UDG), which can be used to control downstream reactions. In one application, we have demonstrated the temporally programmed and pulsed decoration of DNA nanostructures. In the second one, we showed the design of sequentially appearing and self-erasing DNA-based patterns.

Due to the fact that nucleic acid SDRs are the key processes of dynamic DNA nanotechnology,^41−44^ the possibility to achieve temporally programmed pulse outputs could find different applications. For example, being able to intermittently activate DNA-based signaling with specific temporal features could be used to improve multiplexing in DNA-based imaging systems (such as DNA paint)^45^ or to temporally control inputs/outputs in DNA-encoded systems with possible applications in DNA computing and drug-delivery.^46−48^ Finally, inspired by the temporal program of gene expression, our strategy can be used to control over time the pulse activation of synthetic genes for different synthetic biology applications, including diagnostic, therapeutic, and biofuel production.^49,50^

## Experimental Section

### Chemicals

All reagent-grade chemicals, including [DEPC-treated water, MgCl_2_, Trizma hydrochloride, ethylenediaminetetraacetic acid (EDTA), NaCl, 1,4-Dithiothreitol(DTT)] were purchased from Sigma-Aldrich (Italy) and used without further purifications.

### Enzymes

UDG and RNase H recombinant were purchased from New England Biolabs (Beverly, MA, USA). Before use, RNase H was previously activated by incubation for 1 h at 37 °C in 50 mM Tris-HCl, 50 mM KCl, and 3 mM MgCl_2_, in the presence of 50 mM DTT at pH 8.0.

### Oligonucleotides

Oligonucleotides employed in this work were synthesized, labeled, and HPLC-purified by Metabion International AG (Planegg, Germany) and used without further purification. The DNA oligonucleotides were dissolved in phosphate buffer 50 mM, pH 7.0, and stored at −20 °C until use. The RNA oligonucleotides were dissolved in DEPC-treated water and stored at −20 °C until use. All the sequences of the different systems are reported in the Supporting Information document.

### Fluorescence Experiments

Fluorescence kinetic measurements were carried out on a Tecan Infinite M Nano+ plate reader using the top reading mode with black, flat bottom nonbinding 96-well plates, and a 100 μL final volume. The concentrations employed and buffer conditions are reported in the legend of each figure or in the Supporting Information. Detailed procedures employed in the different experiments are reported in the Supporting Information document.

### Fluorescence Microscopy Experiments

The experimental conditions for pulse decoration of tubular structures are detailed in the Supporting Information. Briefly, the DNA nanostructures were prepared as reported elsewhere (see SI, Section 1.7). A solution of DNA nanostructures (0.1 μM) was incubated with the target strand (0.05 μM) and the output strand (0.1 μM) and with different concentrations of RNase H. After the addition of the RNA input strand (150 nM), an aliquot of the sample was imaged at different times using an Axio Observer 7.

## Supporting Information Available

Oligonucleotides sequences used; kinetic model, curve fitting, and control and supporting experiments (PDF)
Sequentially-appearing and self-erasing DNA patterns (MP4)
