# Supplementary material for: Timed Pulses in DNA Strand Displacement Reactions
Source: J Am Chem Soc. 2023 Sep 15;145(38):20968–74. doi: 10.1021/jacs.3c06664 (PMC10540199; doi:10.1021/jacs.3c06664)
Supplement: Supplementary file 1 — ja3c06664_si_001.pdf [file ja3c06664_si_001.pdf]

Supporting Information

Timed Pulses in DNA strand displacement reactions

Juliette Bucci,[1] Patrick Irmisch,[2] Erica Del Grosso,[1] Ralf Seidel,[2] and Francesco Ricci,[1,*]

[1]Department of Chemical Sciences and Technologies, University of Rome, Tor Vergata, Via della Ricerca Scientifica, 00133, Rome, Italy.
[2]Molecular Biophysics Group, Peter Debye Institute for Soft Matter Physics, Universität Leipzig, 04103 Leipzig, Germany;

Table of contents

1.Experimental Procedures 3

Oligonucleotides 3

Fluorescence experiments 6

Kinetic measurements 6

Fluorescence microscopy 7

Kinetic measurements 7

2. Kinetic modelling and curve fitting 8

2a. General considerations 8

2b. Curve fitting 9

2c. Rate model for pulse-DNA strand displacement reactions with programmable temporal delay using RNase H 9

2d. Rate model for pulse-DNA strand displacement reactions with programmable temporal delay using UDG and RNase H 12

3.Supplementary figures 14

References 28

1. Experimental Procedures

Oligonucleotides

Sequences for achieving timed pulses in strand displacement reaction (SDR) are listed below.

System 1: Timed pulses in SDRs using RNase H (Figure 2,4,6)

**Name**	**Sequence**
Target	5'- ATC T**TC ATA CAC GTT GCT AG**G TCT CGC TAT CAG GAT CTA T- BHQ-2 -3'
Output	5'- Cy3 - ATA GAT CCT GAT AGC GAG AC -3'
RNA-blocker	5'- CUA GCA ACG UGU AUG A -3'
RNA input	5'- AUA GAU CCU GAU AGC GAG ACC UAG C -3'

In this system the target duplex is composed by the output and target sequences, that are respectively conjugated to a fluorophore (Cy3) and a quencher (BHQ-2). The bold bases in the target strand represent the blocker-binding region, while the underlined bases indicate the toehold domain. The toehold-binding domain is contained in the RNA-blocker and RNA input and is represented by the underlined bases.

System 2: Timed pulses in SDRs using UDG and RNase H (Figure 3,4,6)

**Name**	**Sequence**
Target	5'- AAC A**CT TCA CAA CTA CAG CT**T CAA TTC AGG ACA ATC GGC T- BHQ-2 -3'
Output	5'- Cy5 - AGC CGA TTG TCC TGA ATT GA -3'
Uracil-blocker	5'- AGC (2- Deoxyuridine)G(2- Deoxyuridine) AG(2- Deoxyuridine) TG(2- Deoxyuridine) GAA G -3'
RNA Input	5'- AGC CGA UUG UCC UGA AUU GAA GCU G -3'

In this second system the target duplex is composed by the output and target sequences, that are respectively conjugated to a fluorophore (Cy5) and a quencher (BHQ-2). The bold bases in the target strand represent the blocker-binding region, while the underlined bases indicate the toehold domain. The toehold-binding domain is contained in the RNA-blocker and RNA input and is represented by the underlined bases.

System 3: Timed pulse decoration of DNA nanostructures using RNase H (Figure 5,S11,S12)

DNA tile sequences

**Name**	**Sequence**
S1	5'-CTC AGT GGA CAG CCG TTC TGG AGC GTT GGA CGA AAC T-3'
S2	5'- *GTC TG*G TAG AGC ACC ACT GAG *AGG TA*-3'
S3	5'- TCC AGA ACG GCT GTG GCT AAA CAG TAA CCG AAG CAC CAA CGC-3'
S3_anchor1	5'- TCC AGA ACG GCT GTG GCT AAA CAG TAA CCG AAG CAC CAA CGC T**TT TTT TTT TTT GTG AAT ATA AGA TCG AAC G**-3'
S4	5'-*CAG AC*A GTT TCG TGG TCA TCG *TAC CT*-3
S5	5'-CGA TGA CCT GCT TCG GTT ACT GTT TAG CCT GCT CTA C-3'

The DNA tiles are composed by the sequences listed above. The italics bases of S2 and S4 represent the sticky end portions that allow the self-assembly into DNA tubular structures. While the bold bases of the S3_anchor1 strand indicate the anchor portion that allows the decoration of the DNA-tiles.

DNA nanostructure decorating sequences

Target	5'- ATC T**TC ATA CAC GTT GCT AG**G TCT CGC TAT CAG GAT CTA TTT T*CG TTC GAT CTT ATA TTC ACA*
Output	5'- ATA GAT CCT GAT AGC GAG AC -3'
RNA-blocker	5'- CUA GCA ACG UGU AUG A -3'
RNA Input	5'- Cy5- AUA GAU CCU GAU AGC GAG ACC UAG C -3'

The DNA/RNA strands reported above are used for achieving the timed pulse decoration of DNA nanostructures. The italic bases on the target strand represent the portion able to interact with the S3_anchor 1 of the DNA tile. Also in this case the target duplex is composed by the output and target sequences. The bold bases in the target strand represent the blocker-binding region, while the underlined bases in the target, in the RNA-blocker and in the RNA input strand represent the toehold and toehold-binding domains.

System 4: Timed pulse decoration of DNA nanostructures using RNase H and UDG (Figure S13)

DNA tile sequences

**Name**	**Sequence**
S1	5'-CTC AGT GGA CAG CCG TTC TGG AGC GTT GGA CGA AAC T-3'
S2	5'- *GTC TG*G TAG AGC ACC ACT GAG *AGG TA*-3'
S3	5'- TCC AGA ACG GCT GTG GCT AAA CAG TAA CCG AAG CAC CAA CGC-3'
S3_anchor2	5'- TCC AGA ACG GCT GTG GCT AAA CAG TAA CCG AAG CAC CAA CGC T**TT TTT TTT TTT CTA GAA TTT CCT ACT CGA T**-3'
S4	5'-*CAG AC*A GTT TCG TGG TCA TCG *TAC CT*-3'
S5	5'-CGA TGA CCT GCT TCG GTT ACT GTT TAG CCT GCT CTA C-3'

The DNA tiles are composed by the sequences listed above. The italics bases of S2 and S4 represent the sticky end portions that allow the self-assembly into DNA tubular structures. While the bold bases of the S3_anchor2 strand indicate the anchor portion that allows the decoration of the DNA-tiles.

DNA nanostructure decorating sequences

Target	5'- AAC A**CT TCA CAA CTA CAG CT**T CAA TTC AGG ACA ATC GGC TTT T*AT CGA GTA GGA AAT TCT AGA* -3'
Output	5'- AGC CGA TTG TCC TGA ATT GA -3'
Uracil-blocker	5'- AGC (2- Deoxyuridine)G(2- Deoxyuridine) AG(2- Deoxyuridine) TG(2- Deoxyuridine) GAA G -3'
RNA Input	5'- Cy3- AGC CGA UUG UCC UGA AUU GAA GCU G -3'

The DNA/RNA strands reported above are used for the timed pulse decoration of DNA nanostructures. The italic bases on the target strand represent the portion able to interact with the S3_anchor 2 of the DNA tile. Also in this case the target duplex is composed by the output and target sequences. The bold bases in the target strand represent the blocker-binding region,

while the underlined bases in the target, uracil-blocker and RNA input strand represent the toehold and toehold-binding domains.

Fluorescence experiments

Kinetic measurements

Fluorescence kinetic measurements were carried out on a Tecan Infinite M Nano+ plate reader using the top reading mode with black, flat bottom non-binding 96-well plates. The working wavelengths were set to λ_{exc} = 550 (±9) nm and λ_{emi} =585 (±20) nm for the Cy3 labeled oligonucleotides, λ_{exc} = 645 (±9) nm and λ_{emi}= 680 (±20) nm for the Cy5 labeled oligonucleotides.

Pulse delay strand displacement reactions

The experiments shown in Figures 2,3 were performed at 30 °C in 20 mM Tris-HCl buffer, 10 mM $MgCl_2$, 1 mM EDTA, pH 8.0. The initial target duplex (50 nM) was formed by incubating at 90°C for 2 minutes target and output strands in presence of the desired concentration of blocker strands. Only for the system with the RNA-blocker the incubation was performed at 60°C. After 30 minutes the preformed duplex with blocker was transferred (100 μL) to a 96-well plates where 10 mM DTT and 150 nM RNA input strand were added. After the stabilization of the signal, the corresponding enzyme was added in the wells at the desired concentration and the fluorescence intensity was recorded over time.

Orthogonal timed pulses SDRs

The experiments shown in Figure 4, S10 were performed at 30 °C in 20 mM Tris-HCl buffer, 10 mM $MgCl_2$, 1 mM EDTA, pH 8.0. The strands forming the target duplex for each pulse delay strand displacement reactions were incubated together at 60°C for 2 minutes, using equimolar concentrations and the desired concentration of blockers. After 30 minutes the preformed complexes were transferred (100 μL) to 96-well plates where DTT (10 mM) and the two RNA input strands (150 nM) were added. After the stabilization of the signal, the two enzymes (at the desired concentration) were added into the wells at the same time and the two different fluorescence intensities were recorded over time.

Pattern pulse delay strand displacement reactions

The experiments shown in Figure 6, S14 were performed at 30 °C in 20 mM Tris-HCl buffer, 10 mM $MgCl_2$, 1 mM EDTA, pH 8.0. The strands forming the target duplex for each enzyme-controlled strand displacement system were incubated separately at 60°C for 2 minutes, using

equimolar concentrations of the two relevant strands (target and output) and the desired concentration of blockers. After 30 minutes, the different preformed duplexes were combined together (50 µL of each sample) into the 96-well plates, where DTT (10 mM) and the two RNA input strands (150 nM) were added. After the stabilization of the signal, the two enzymes (at the desired concentration) were added into each well at the same time and the two different fluorescence intensity were recorded over time. As a fluorescent reference signal we have employed for system-1 a well containing the free cy3-output strand at 50 nM and for system-2 another well containing the free cy5-output strand at 50 nM.

Fluorescence microscopy

Pulse delayed decoration of DNA nanostructure

For all the experiments shown in Figure 5,S11-13 the DNA nanostructures were prepared in H_2O/Mg^{2+} (12.5 mM $MgCl_2$) by mixing S1, S2, S4 and S5 tile strands at 5 µM final concentration, while S3 strand and corresponding S3_anchor strand (that allows the decoration of the DNA nanostructure) were used at 2.5 µM, in order to have the 50% of the DNA tile containing the anchor strand. DNA nanostructures were annealed using a Bio-Rad Mastercycler Gradient thermocycler PCR machine by heating the samples to 90°C, and cooling to 20°C at a constant rate (1°C / 5min).

In order to decorate the DNA nanostructures, a solution containing the target (0.5 µM), the output (1 µM) and the desired concentration of blocker strand (RNA or Uracil-modified strand), was incubated at 60°C. After 30 minutes an aliquot of this solution was mixed with an aliquot of the previously annealed nanostructures (5 µM) to achieve a final concentration of 0.1 µM of DNA nanostructures, 0.05 µM of target strand and 0.1 µM of output strand.

Finally to demonstrate the pulse delay decoration of the DNA nanostructures, a solution of the decorated DNA nanostructures (100 nM) was incubated with RNase H at the desired concentrations. After the addition of the RNA input strand (150 nM), an aliquot of the sample was imaged at different times.

Kinetic measurements

DNA-based nanostructures were imaged using an Inverted microscope Axio Observer 7. The emitted photons were collected by a 100x oil immersion objective (EC Plan-Neo Fluor) and a monochrome camera (Axiocam 305). All the samples containing DNA nanostructures were imaged at 100 nM tiles concentration in the corresponding experimental condition. A single

drop (1.5 µL) of the sample was deposited on a microscope coverslip and covered with a coverslip (Menzel-Glaser; thickness: 0.13 to 0.16 mm; size: 18x18 mm).

Images were taken using 90 HE LED filters with specific excitation wavelengths (385 nm, 475 nm, 555 nm, and 630 nm) and a QBS filter (405 nm + 493 nm + 575 nm + 653 nm) for simultaneous excitation. Additionally, emission was detected using a QBP filter (425/30 nm + 514/30 nm + 592/30 nm + 709/100 nm). The exposure time for image acquisition was set at 10000 ms. Fluorescence microscopy images were processed using ZEN 2 lite (ZEISS) software.

Microscopy data processing

SPIP Software –Scanning Probe Image Processor – by Image Metrology/Digital Surf (www.imagemet.com) was employed for the pixel analysis of the fluorescence microscopy images, by counting the number of pixels in a region of interest. Branched structures, aggregates, and structure lengths less than 0.5 µm were removed from the dataset analysis using SPIP threshold parameters.

2. Kinetic modelling and curve fitting

Kinetic modeling and curve fitting

2a. General considerations

In order to understand the experimental results obtained in this study, we devised simplified reaction schemes that comprise solely the key kinetic steps, while disregarding rapid forward reactions and slow reverse reactions.[1,2] Specifically, our models treated strand displacement reactions as single-step, irreversible processes. For enzymatic reactions, we assumed protein activity to occur under saturation conditions (i.e. significantly higher concentrations of proteins and substrates compared to the dissociation constant K_D). All reaction steps considered in our models were assumed to follow first or second-order kinetics. By numerically integrating the resulting sets of differential equations derived from the reaction schemes, we obtained time-dependent concentration profiles for the various reaction species.

The conventional strand displacement reactions (Figure S3) were assumed to exhibit simple second-order kinetics according to the following scheme with the displacement rate constant k_{displ}:

$$AB + C \xrightarrow{k_{\text{displ.}}} AC + B. \qquad (1)$$

In this scheme, the initial target duplex (*AB*), composed of the target strand (*A*) and the output strand (*B*), reacts with an input strand (*C*), leading to the release of the output strand.

The set of rate equations, that describe the conventional strand displacement are:

$$\frac{d[AB]}{dt} = \frac{d[C]}{dt} = \quad - k_{\text{displ.}}[AB][C], \qquad (2)$$

$$\frac{d[AC]}{dt} = \frac{d[B]}{dt} = \quad + k_{\text{displ.}}[AB][C]. \qquad (3)$$

The resulting time profiles of the output concentration were linearly related to the measured fluorescence signal (F) via a scaling factor (S):

$$F = S[B]. \qquad (4)$$

2b. Curve fitting

All curve fitting was performed using custom Python (version Python 3.8.8) scripts. The scripts utilize the "odeint" function for numerical integration of a set of ordinary differential equations. The resulting solution was then fitted to the observed kinetics using the "curve_fit" function for nonlinear least-squares optimization. Both functions are included in the SciPy package.[3]

2c. Rate model for pulse-DNA strand displacement reactions with programmable temporal delay using RNase H

The experimental data obtained for the pulse-DNA strand displacement reactions with programmable temporal delay employing RNase H (Figure 2) was modeled according to the kinetic scheme shown in Figure S4.

In the starting configuration of this scheme, the blocker strands (*O*) are bound to the toehold region of the initial target-output duplex (*AB*) to form the complex *ABO*. Degradation of the blocker strands being bound to the output-target complex was assumed to occur under pseudo-first-order conditions with rate constant $\mathrm{k}_{\mathrm{deg}}^{1}$ such that it is described by the reaction:

$$ABO \xrightarrow{\mathrm{k}_{\mathrm{deg}}^{1}} AB + R, \qquad (5)$$

during which unblocked output-target complexes *AB* as well as degraded blocker fragments *R* are produced. After the release of the blocker strand, the output-target duplexes can either rebind another blocker strand with the rate constant k_{bind} (as long as intact blockers are in solution):

$$AB + O \xrightarrow{k_{\text{bind}}} ABO, \qquad (6)$$

or perform strand displacement with the input strand *C* to release the output strand *B* according to:

$$AB + C \xrightarrow{k_{\text{displ.}}} AC + B. \tag{7}$$

After the release of the output strand, the RNA input within the input-target heteroduplex is degraded by RNase H, which was again assumed to occur under pseudo-first-order conditions with rate constant $\mathrm{k}_{\text{deg}}^{2}$ according to:

$$AC \xrightarrow{\mathrm{k}_{\text{deg}}^{2}} A + R, \tag{8}$$

during which free target strands *A* as well as degraded blocker fragments *R* are produced.

Given pseudo-first-order conditions, $\mathrm{k}_{\text{deg}}^{1}$ and $\mathrm{k}_{\text{deg}}^{2}$ were generally assumed to be proportional to the enzyme concentration [*E*]. For convenience, we took the enzyme concentration given in U/ml as a dimensionless scaling factor for $\mathrm{k}_{\text{deg}}^{1}$ and $\mathrm{k}_{\text{deg}}^{2}$. This allows expressing the degradation rate constants as:

$$\mathrm{k}_{\text{deg}}^{1} = \mathrm{k}_{E}^{1}[E], \tag{9}$$

$$\mathrm{k}_{\text{deg}}^{2} = \mathrm{k}_{E}^{2}[E], \tag{10}$$

where k_{E}^{1} and k_{E}^{2} are the degradation rate constants at 1 U/ml. Of note, significant inhibition of the enzyme activity was not observed when modeling the data.

The free target can then either rebind another input strand or the output strand again:

$$A + B \xrightarrow{k_{\text{bind}}} AB, \tag{11}$$

$$A + C \xrightarrow{k_{\text{bind}}} AC. \tag{12}$$

For simplicity of the reaction scheme and to avoid overfitting of the reactions kinetics, we applied a single value for the rate constants that describe the hybridization between complementary strands (k_{bind} in Equations 6,11,12). Considering values of $10^{6} - 10^{7}/Ms$ obtained for the hybridization rate constant between secondary structure-free DNA oligonucleotides at similar conditions the timescale of rebinding between complementary strands (at the applied concentrations) can be expected to be much faster compared to the observed output pulse signals. [4-6] Therefore, the rebinding rate constant k_{bind} is not an exact representation for the sequence dependent association rate constant of DNA oligonucleotides, but rather an estimate for the hybridization rate constant of two strands within our simplified reaction scheme to reproduce the observed behaviour.

The set of differential equations describing the pulse-DNA strand displacement reactions with programmable temporal delay using RNase H is then given by:

$$\frac{d[ABO]}{dt} = \quad -\mathrm{k}_{\text{deg}}^{1}[ABO] + k_{\text{bind}}[AB][O], \tag{13}$$

$$\frac{d[AB]}{dt} = \quad + \mathrm{k}_{\mathrm{deg}}^{1}[ABO] - k_{\mathrm{bind}}[AB][O] - \ k_{\mathrm{displ}}[AB][C] + k_{\mathrm{bind}}[A][B], \tag{14}$$

$$\frac{d[O]}{dt} = \quad - k_{\mathrm{bind}}[AB][O], \tag{15}$$

$$\frac{d[C]}{dt} = \quad - \ k_{\mathrm{displ}}[AB][C] - k_{\mathrm{bind}}[A][C], \tag{16}$$

$$\frac{d[AC]}{dt} = \quad + \ k_{\mathrm{displ}}[AB][C] + k_{\mathrm{bind}}[A][C] - \mathrm{k}_{\mathrm{deg}}^{2}[AC], \tag{17}$$

$$\frac{d[A]}{dt} = \quad + \mathrm{k}_{\mathrm{deg}}^{2}[AC] - k_{\mathrm{bind}}[A][B] - k_{\mathrm{bind}}[A][C], \tag{18}$$

$$\frac{d[B]}{dt} = \quad + \ k_{\mathrm{displ}}[AB][C] - k_{\mathrm{bind}}[A][B], \tag{19}$$

$$\frac{d[R]}{dt} = \quad + \mathrm{k}_{\mathrm{deg}}^{1}[ABO] + \mathrm{k}_{\mathrm{deg}}^{2}[AC]. \tag{20}$$

The obtained time course of the free output strands (B) was then related to the measured fluorescence signal.

When fitting the model to experimental data we first obtained the displacement rate constant by performing a measurement in the absence of dissipative conditions and blocker strands (Figure S5) and applying Equations 2-4. Therefore, the ground level (no addition of input strands) was subtracted from the obtained traces. The displacement rate constant for the RNase H based system was determined to be $k_{\mathrm{displ}} = (2.51 \pm 0.02) \cdot 10^{4}/Ms$.

Then we performed a global fit of the blocker and enzyme concentration-dependent series (Figure 2b,d) to obtain the other rate constants of the set of differential equations (k_{bind}, k_{E}^{1} and k_{E}^{2}). To this end, we first subtracted the ground level (150 nM Blocker strands and 0 U/ml enzyme) from all traces, and normalized the signal against the maximum amplitude during the obtained time course. To relate the model curves to the obtained fluorescence trajectories, we normalized the time-dependent model concentration [*B*] by its maximum concentration.

For the rebinding rate constant $k_{\mathrm{bind}} = (9.32 \pm 0.01) \cdot 10^{5}/Ms$, a single value was taken for all traces. To account for small variations in enzyme concentration/activity, which are mainly caused by pipetting errors, the activity rate constants k_{E}^{1} and k_{E}^{2} were individual fit parameters for each trace (Figure S6). To predict the reaction maximum amplitude times, as a function of blocker or enzyme concentration (Figure 2c,e), we used the average values of the activity rate constants (Figure S6, $\mathrm{k}_{E}^{1} = (18 \pm 4) \cdot 10^{-4}/s$ and $\mathrm{k}_{E}^{2} = (8 \pm 2) \cdot 10^{-4}/s$). This 95% confidence intervals was determined by simulating the reaction for a sample set of 1000 random parameter combinations. Each parameter set was generated using a multivariate Gaussian distribution based on the estimated covariance matrix provided by the fit routine.

Afterward, the confidence interval was selected as the range between the 2.5 and 97.5 percentiles, representing the lower and upper boundaries, respectively.

2d. Rate model for pulse-DNA strand displacement reactions with programmable temporal delay using UDG and RNase H

The experimental data obtained for the pulse-DNA strand displacement reactions with programmable temporal delay that employs UDG to degrade a Uracil blocker and RNase H to degrade an RNA input (Figure 3) was modeled according to the kinetic scheme shown in Figure S4. This reaction scheme is similar to the system that employs solely RNase H, but expanded by a release step of the blocker strands and the activity of UDG on single-stranded DNA.

Since the observed pulse signals exhibited a significant delay in the presence of high enzyme and low blocker concentrations, we considered that the creation of abasic sites within the Uracil blocker does not lead to an instant but a rather gradual strand release. This expands Equation 5 to:

$$ABO \xrightarrow{\mathrm{k}_{\mathrm{deg}}^{1}} ABO^{*}, \tag{21}$$

$$ABO^{*} \xrightarrow{k_{\mathrm{release}}} AB + R. \tag{22}$$

Furthermore, we considered that UDG has a significant activity on single-stranded DNA in addition to its activity on double-stranded DNA.[7,8] We incorporated this feature by including the pathway:

$$O \xrightarrow{\gamma \mathrm{k}_{\mathrm{deg}}^{1}} R, \tag{23}$$

where we applied the rate constant for double-stranded degradation, but multiplied it with a scaling factor γ to account for the different activity on single-stranded compared to double-stranded DNA.

The set of differential equations describing the pulse-DNA strand displacement reactions with programmable temporal delay using UDG to degrade Uracil blockers and RNase H to degrade the RNA input is then given by:

$$\frac{d[ABO]}{dt} = -\mathrm{k}_{\mathrm{deg}}^{1}[ABO] + k_{\mathrm{bind}}[AB][O], \tag{24}$$

$$\frac{d[ABO^{*}]}{dt} = +\mathrm{k}_{\mathrm{deg}}^{1}[ABO] - k_{\mathrm{release}}[ABO^{*}], \tag{25}$$

$$\frac{d[AB]}{dt} = +k_{\mathrm{release}}[ABO^{*}] - k_{\mathrm{bind}}[AB][O] - k_{\mathrm{displ}}[AB][C] + k_{\mathrm{bind}}[A][B], \tag{26}$$

$$\frac{d[O]}{dt} = -k_{\text{bind}}[AB][O] - \gamma \mathrm{k}_{\text{deg}}^{1}[O], \quad (27)$$

$$\frac{d[C]}{dt} = -k_{\text{displ}}[AB][C] - k_{\text{bind}}[A][C], \quad (28)$$

$$\frac{d[AC]}{dt} = +k_{\text{displ}}[AB][C] + k_{\text{bind}}[A][C] - \mathrm{k}_{\text{deg}}^{2}[AC], \quad (29)$$

$$\frac{d[A]}{dt} = +\mathrm{k}_{\text{deg}}^{2}[AC] - k_{\text{bind}}[A][B] - k_{\text{bind}}[A][C], \quad (30)$$

$$\frac{d[B]}{dt} = +k_{\text{displ}}[AB][C] - k_{\text{bind}}[A][B], \quad (31)$$

$$\frac{d[R]}{dt} = +k_{\text{release}}[ABO^*] + \mathrm{k}_{\text{deg}}^{2}[AC] + \gamma \mathrm{k}_{\text{deg}}^{1}[O]. \quad (32)$$

The obtained time course of the free output strands (B) was then related to the measured fluorescence signal.

The general fitting approach was the same as before. We first determined the displacement rate constant $k_{\text{displ}} = (1.437 \pm 0.003) \cdot 10^3/Ms$ from fitting displacement experiments in the absence of blocker strands (Figure S8) and applying equations 2-4. Therefore, the ground level (no addition of input strands) was subtracted from the obtained traces. For the rebinding rate constant $k_{\text{bind}} = (9.32 \pm 0.01) \cdot 10^5/Ms$, we applied the value obtained from the RNase H based system for all traces.

Then we performed a global fit of the blocker and enzyme concentration-dependent series (Figure 3b,d) to obtain the other rate constants of the set of differential equations (k_{release}, γ, k_E^1 and k_E^2). Analogously to before, we first subtracted the ground level (150 nM Blocker strands and 0 U/ml enzyme) from all traces, and normalized the signal against the maximum amplitude during the obtained time course. To relate the model curves to the obtained fluorescence trajectories, we normalized the time-dependent model concentration [*B*] by its maximum concentration.

For the blocker release rate constant $k_{\text{release}} = (3.109 \pm 0.003) \cdot 10^{-3}/s$ and the scaling parameter for the activity on single-stranded DNA $\gamma = 0.2025 \pm 0.0002$, single values were applied for all traces. Again, the activity rate constants k_E^1 and k_E^2 were individual fit parameters for each trace (Figure S9). To predict the reaction maximum-amplitude times, as a function of blocker or enzyme concentration (Figure 3c,e), we used the average values of the activity rate constants (Figure S9, $\mathrm{k}_E^1 = (11.9 \pm 2.2) \cdot 10^{-4}/s$ and $\mathrm{k}_E^2 = (1.8 \pm 0.3) \cdot 10^{-4}/s$). This 95% confidence intervals was determined by simulating the reaction for a sample set of 1000 random parameter combinations. Each parameter set was generated using a multivariate Gaussian distribution based on the estimated covariance matrix provided by the fit routine.

Afterward, the confidence interval was selected as the range between the 2.5 and 97.5 percentiles, representing the lower and upper boundaries, respectively.

3.Supplementary figures

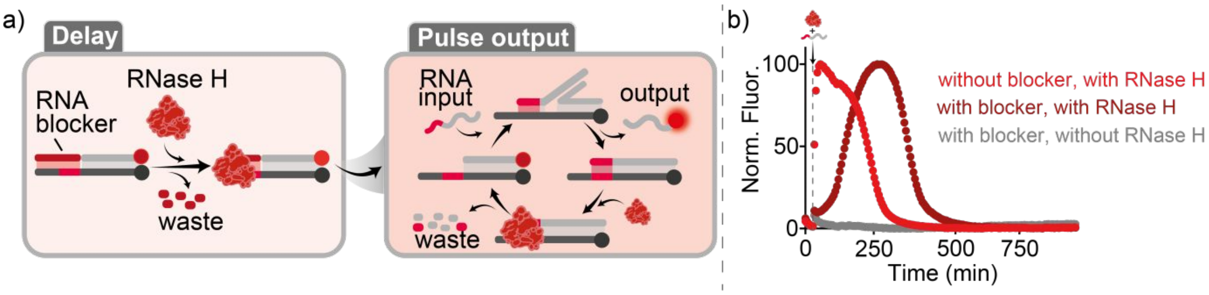

Figure S1.**Timed pulses in SDRs using RNase H**. a) Scheme of the reaction steps. b) Time-course experiments of the SDR carried out in the following conditions: i) without RNase H (grey trace), ii) with blocker strand and RNase H (dark red trace), iii) without blocker strand and in presence of RNase H (light red trace). Experiments shown here were performed in Tris HCl 20 mM, $MgCl_2$ 10 mM, EDTA 1 mM; pH 8.0 at T=30°C using [target duplex] = 50 nM, [input] = 150 nM, [RNA blocker] = 150 nM and [RNase H] = 0.3 U/mL.

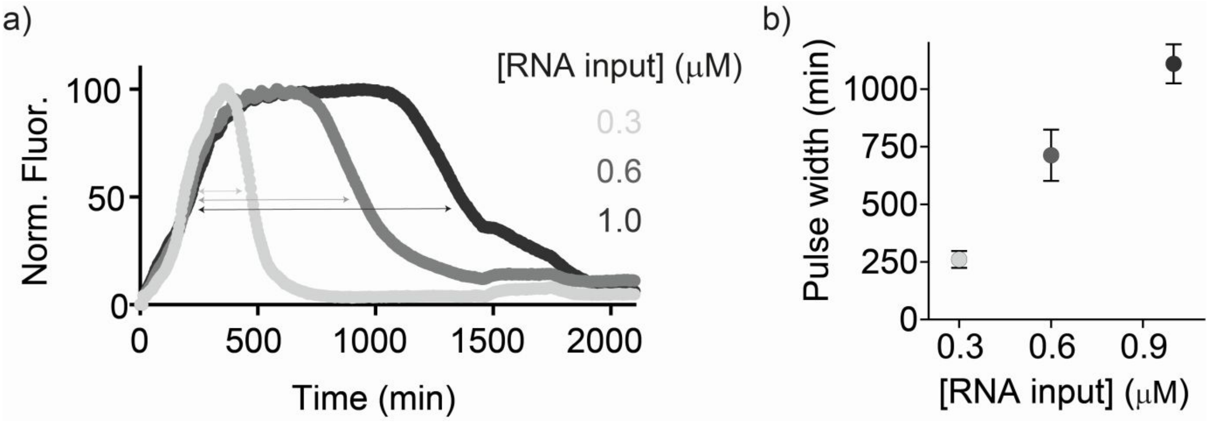

Figure S2. **Pulse width modulation**. a) Time-course experiments of pulse SDR using different concentrations of RNA input (from 0.3 μM to 1.0 μM) b) Pulse width values (defined as the amplitude at 50% of the SDR signal) vs RNA input concentration. Experiments shown here were performed in Tris HCl 20 mM, $MgCl_2$ 10 mM, EDTA 1 mM; pH 8.0 at T=30°C using [target duplex] = 50 nM, [RNA blocker] = 150 nM and [RNase H] = 0.3 U/mL.

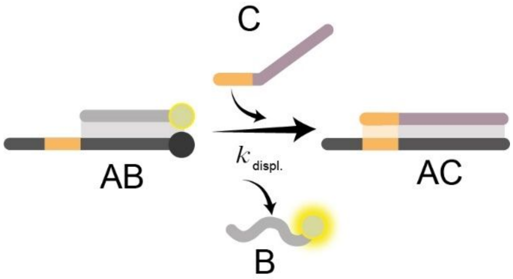

Figure S3. Scheme of a conventional DNA-based strand displacement reactions. An input strand (C) binds to the toehold region (yellow) of the target strand (A) and displaces the pre-hybridized output strand (B).

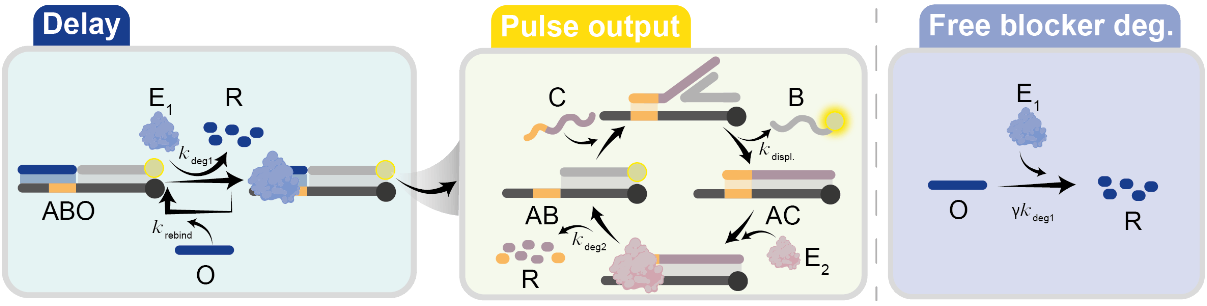

Figure S4. Simple kinetic model for the timed pulses in SDR using different enzymatic reactions. Initially, the blocker strand (O) is bound to the toehold region of the target-output duplex (AB). The presence of the enzyme E_1 leads to the removal of the blocker strand, liberating the toehold region. The free toehold is then either rebound by a new blocker strand or enables the RNA input (C) to carry out the strand displacement reaction to release the output strand. Once the RNA input displaced the output, it is degraded inside the heteroduplex by the enzyme E_2. The free target strand can either rebind another RNA input strand or the output strand again. According to the type of enzyme E_1 employed in parallel it can also happen the degradation of the unbound single-stranded blocker present in solution.

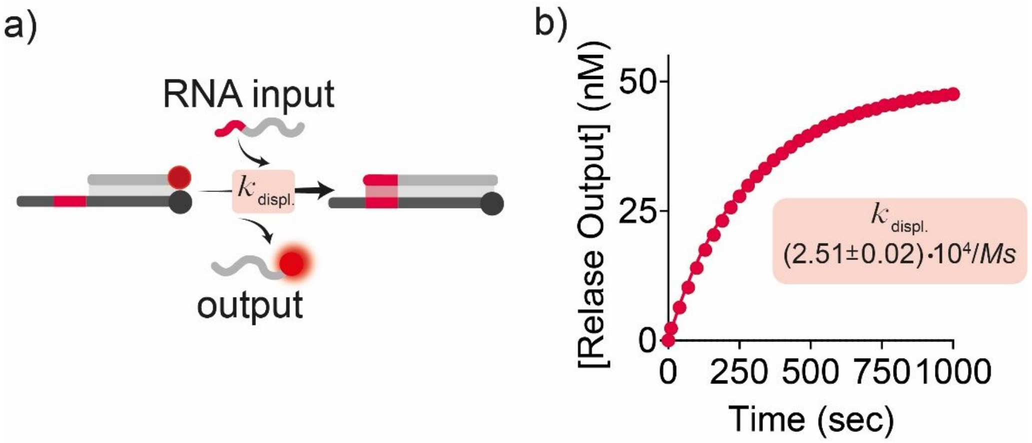

Figure S5. **Conventional strand displacement in the absence of RNA blocker strand**. a) Scheme of the SDR. b) Time trace (filled symbols) of the strand displacement reaction for system 1 in the absence of RNase H and RNA blocker strand. After the addition of the RNA input strand the reaction occurs without delay and run to completion. Solid lines indicate fits to the experimental data based on second-order kinetics. Experiments shown here were performed in Tris HCl 20 mM, $MgCl_2$ 10 mM, EDTA 1 mM; pH 8.0 at T=30°C using [target duplex] = 50 nM and [RNA input] = 150 nM.

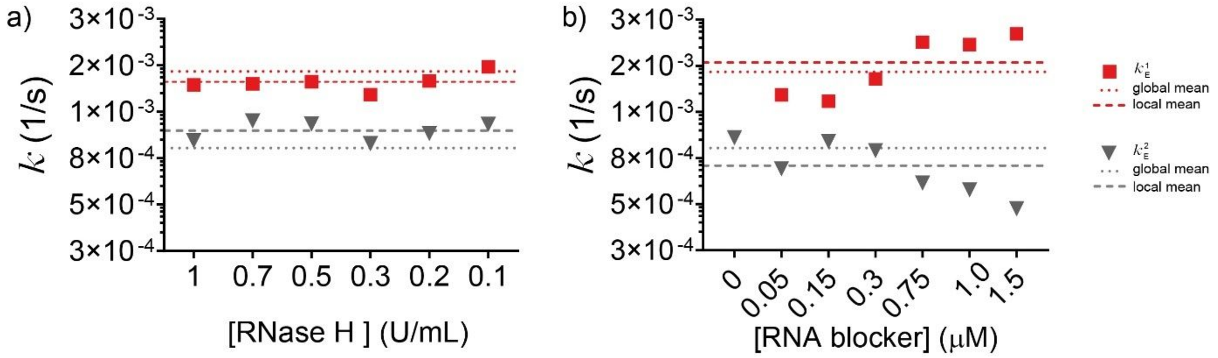

Figure S6. Enzyme activity rate constants for the timed pulses in SDR using RNase H. Rate constants k_E^1 and k_E^2 were obtained from the individual traces from a global fit to the data in Figure 2. k_E^1 and k_E^2 values at different a) RNase H concentrations and b) blocker concentrations. The dashed lines represent the average value for a specific set of measurements, while the dot lines represent the overall average for both sets. The absence of a trend of *k* with respect to the corresponding concentration suggests that variations in the rate constants were primarily caused by errors during the pipetting and mixing process.

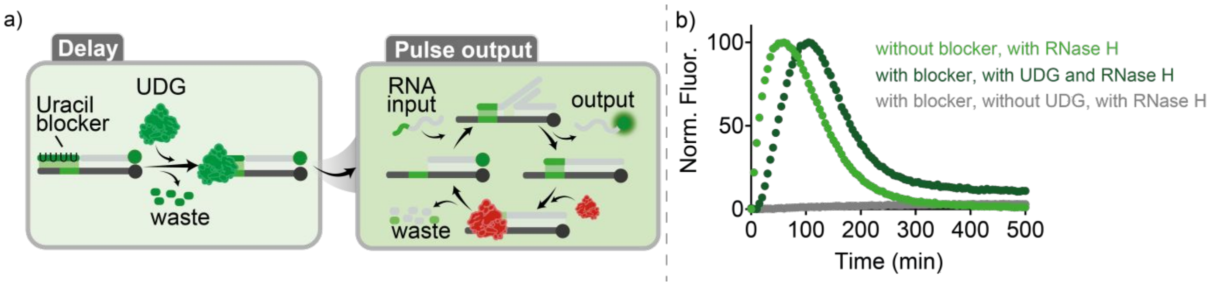

Figure S7. **Timed pulses in SDRs using UDG and RNase H**. a) Scheme of the reaction steps. b) Time-course experiments of the SDR carried out in the following conditions: i) without UDG (grey trace), ii) with uracil blocker strand, UDG and RNase H (dark green trace), iii) without blocker strand in the presence of RNase H (light green trace). Experiments shown here were performed in Tris HCl 20 mM, $MgCl_2$ 10 mM, EDTA 1 mM; pH 8.0 at T=30°C using [target duplex] = 50 nM, [input] = 150 nM, [RNA blocker] = 150 nM, [UDG] = 0.5 U/mL and [RNase H] = 0.5 U/mL.

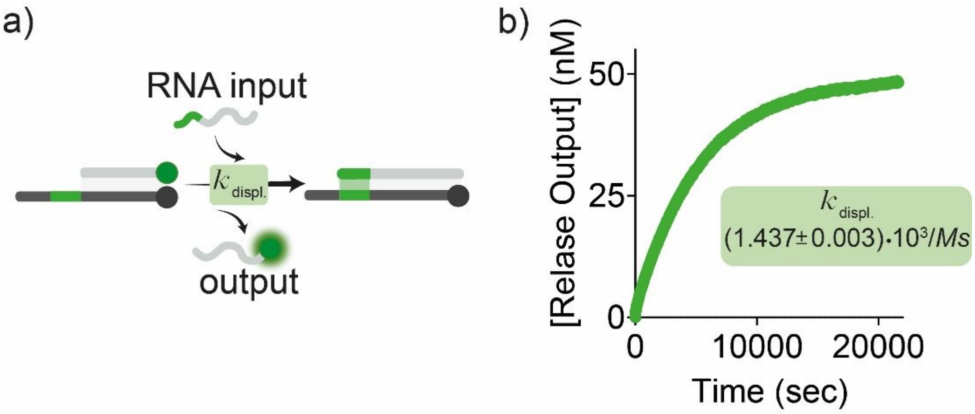

Figure S8. **Conventional strand displacement in the absence of uracil blocker strand**. a) Scheme of the SDR. b) Time trace (filled symbols) of the strand displacement reaction for system 2 in the absence of UDG, RNase H and uracil blocker strand. After the addition of the RNA input strand, the reaction occurs without delay and run to completion. Solid lines indicate fits to the experimental data based on second-order kinetics. Experiments shown here were performed in Tris HCl 20 mM, $MgCl_2$ 10 mM, EDTA 1 mM; pH 8.0 at T=30°C using [target duplex] = 50 nM and [input] = 150 nM.

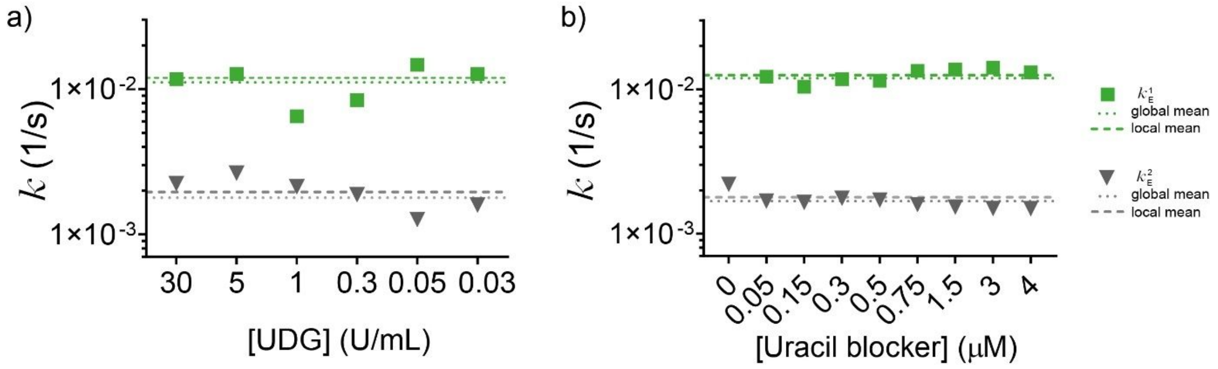

Figure S9. Enzyme activity rate constants for timed pulses in SDR using the RNase H and UDG enzymes. Rate constants k_E^1 and k_E^2 were obtained from the individual traces from a global fit to the data in Figure 3. k_E^1 and k_E^2 values at different a) UDG concentrations. and b) blocker concentrations. The dashed lines represent the average value for a specific set of measurements, while the dote lines represent the overall average for both sets. The absence of a trend of *k* with respect to the corresponding concentration suggests that variations in the rate constants were primarily caused by errors during the pipetting and mixing process.

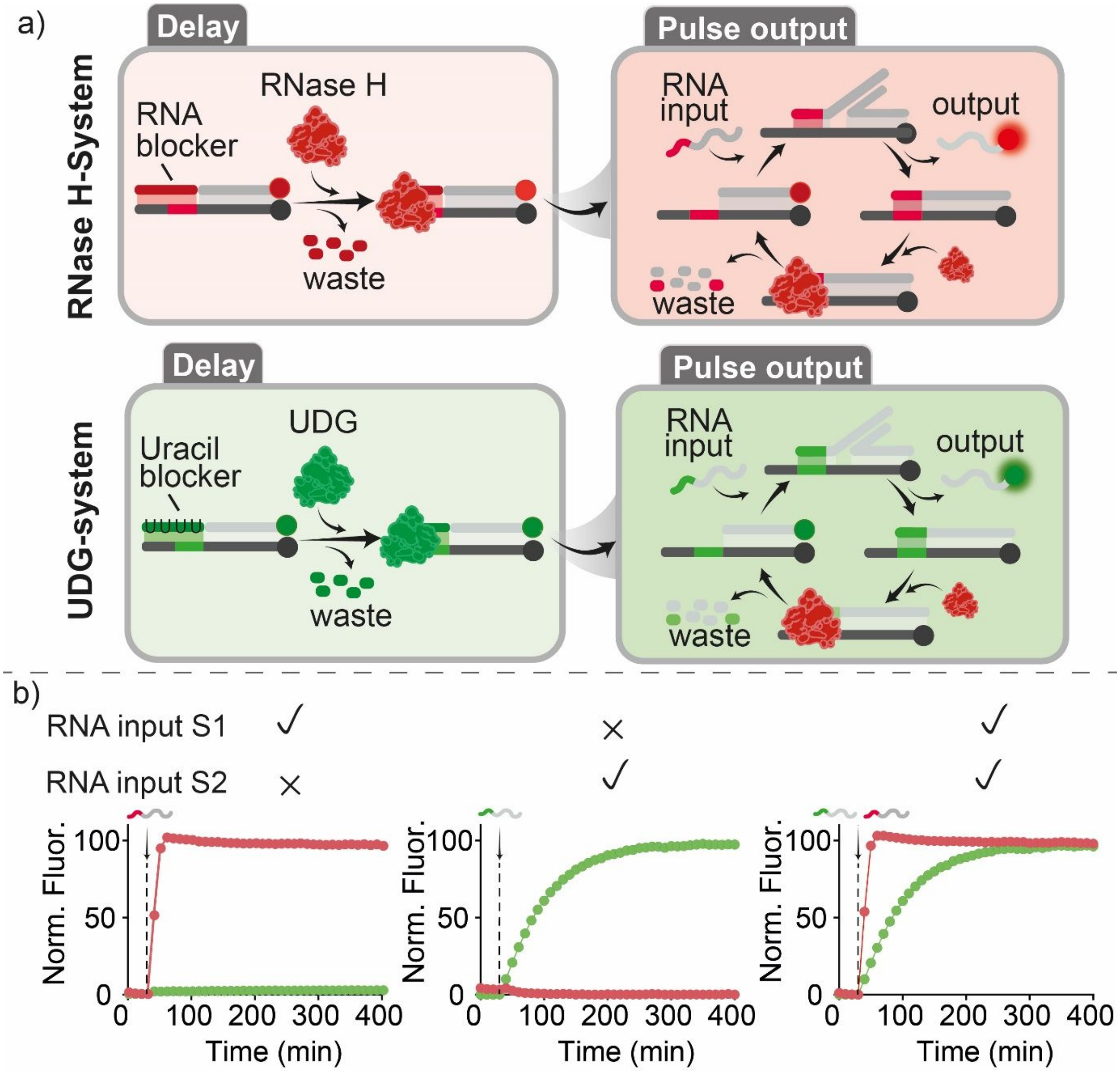

Figure S10. Orthogonality of the two strand displacement systems in the absence of the blocker strand. a) Scheme showing the two different SDR systems, each controlled by a different input strand, a different blocker strand and labeled with a different fluorophore/quencher pair for orthogonal temporal control in the same solution. b) Time-course experiments of the SDRs carried out in the presence of both the target duplexes and activated with only a single RNA input strand. Displacement is only obtained for the system with the corresponding specific input demonstrating full orthogonality. Experiments shown here were performed in Tris HCl 20 mM, $MgCl_2$ 10 mM, EDTA 1mM; pH 8 at T=30°C. [Target duplex] = 50 nM, [RNA input S1] = 150 nM, [RNA input S2] = 150 nM.

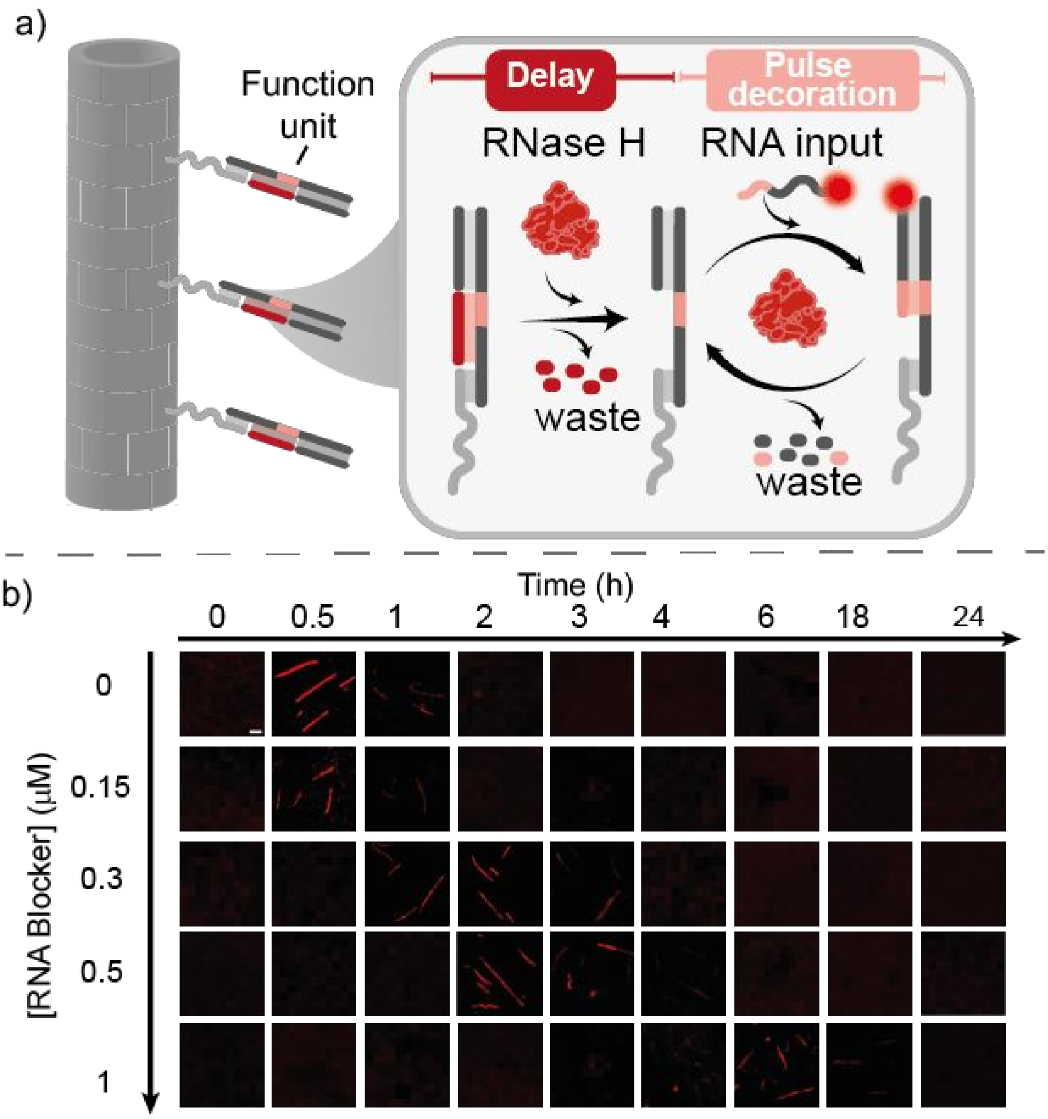

Figure S11. Timed pulse decoration of DNA nanostructures at different concentrations of RNA blocker strand. a) Scheme of the reactions on the DNA structure. Each DNA tile displays a 20-nt ssDNA overhang that serves as anchor for the target duplex. An RNA blocker strand and an RNA input strand labelled with a fluorophore tag (Cy3) were used together with RNase H as the degradation enzyme. b) Fluorescence microscopy images of the DNA structures after RNase H addition, at different times and at different concentrations of the RNA blocker strand. Scale bar 2 μm. The experiments shown in this figure were performed in Tris HCl 20 mM, $MgCl_2$ 10 mM, EDTA 1 mM, pH 8.0 at T=30°C, using 100 nM of assembled structures, [target strand] = 50 nM, [output strand] = 100 nM, [RNA input strand] = 150 nM, [RNase H] = 3 U/mL and the indicated concentration of the RNA blocker strand.

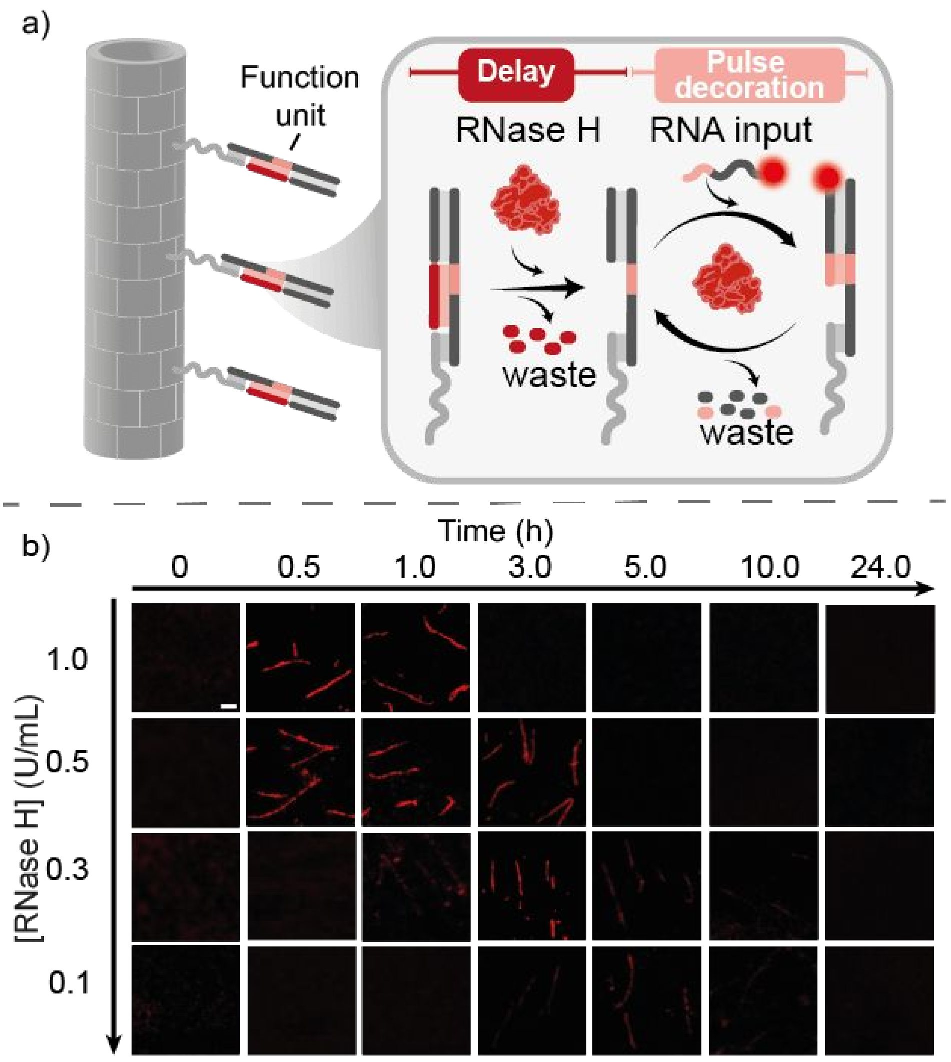

Figure S12. Timed pulse decoration of DNA nanostructures at different concentrations of RNase H. a) Scheme of the reactions on the DNA structure. Each DNA tile displays a 20-nt ssDNA overhang that serves as anchor for the target duplex. An RNA blocker strand and an RNA input strand labelled with a fluorophore tag (Cy3) were used together with RNase H as the degradation enzyme. b) Fluorescence microscopy images of the DNA structures after RNase H addition, at different times and at different concentrations of RNase H. Scale bar 2 µm. The experiments shown in this figure were performed in Tris HCl 20 mM, $MgCl_2$ 10 mM, EDTA 1 mM, pH 8.0 at T=30°C, using 100 nM of assembled structures, [target strand] = 50 nM, [output strand] = 100 nM, [RNA input strand] = 150 nM, [RNA blocker strand] = 150 nM and the indicated concentration of the RNase H.

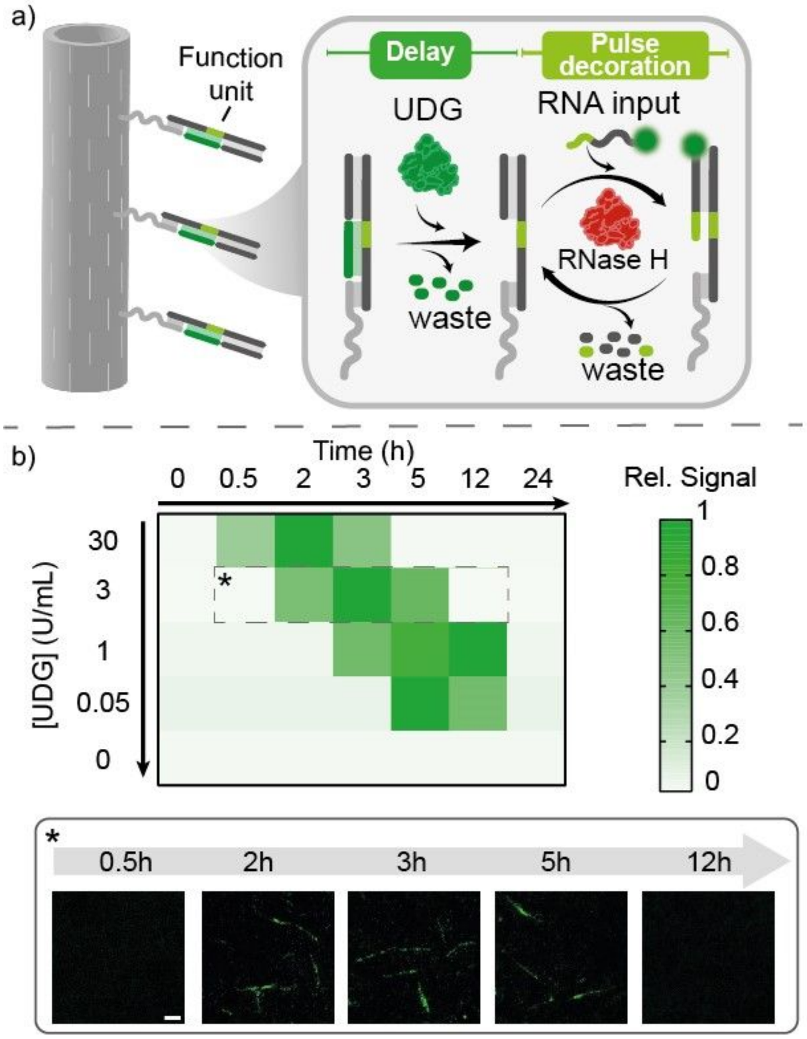

Figure S13. Timed pulse decoration of DNA nanostructures at different concentrations of UDG. a) Scheme of the reactions on the DNA structure. Each DNA tile displays a 20-nt ssDNA overhang that serves as anchor for the target duplex. A Uracil blocker strand and an RNA input strand labelled with a fluorophore tag (Cy5) were used together with UDG as the degradation enzyme. b) Analysis of the pixel intensity of the structures at different concentrations of UDG. and fluorescence microscopy images of the DNA structures after UDG addition, at different times and using 150 nM of the Uracil blocker strand. Scale bar 2 μm. The experiments shown in this figure were performed in Tris HCl 20 mM, $MgCl_2$ 10 mM, EDTA 1 mM, pH 8.0 at T=30°C, using 100 nM of assembled structures, [target strand] = 50 nM, [output strand] = 100 nM, [RNA input strand] = 150 nM, [Uracil blocker strand] = 150 nM, [RNase H] = 3.0 U/mL and the indicated concentration of the UDG.

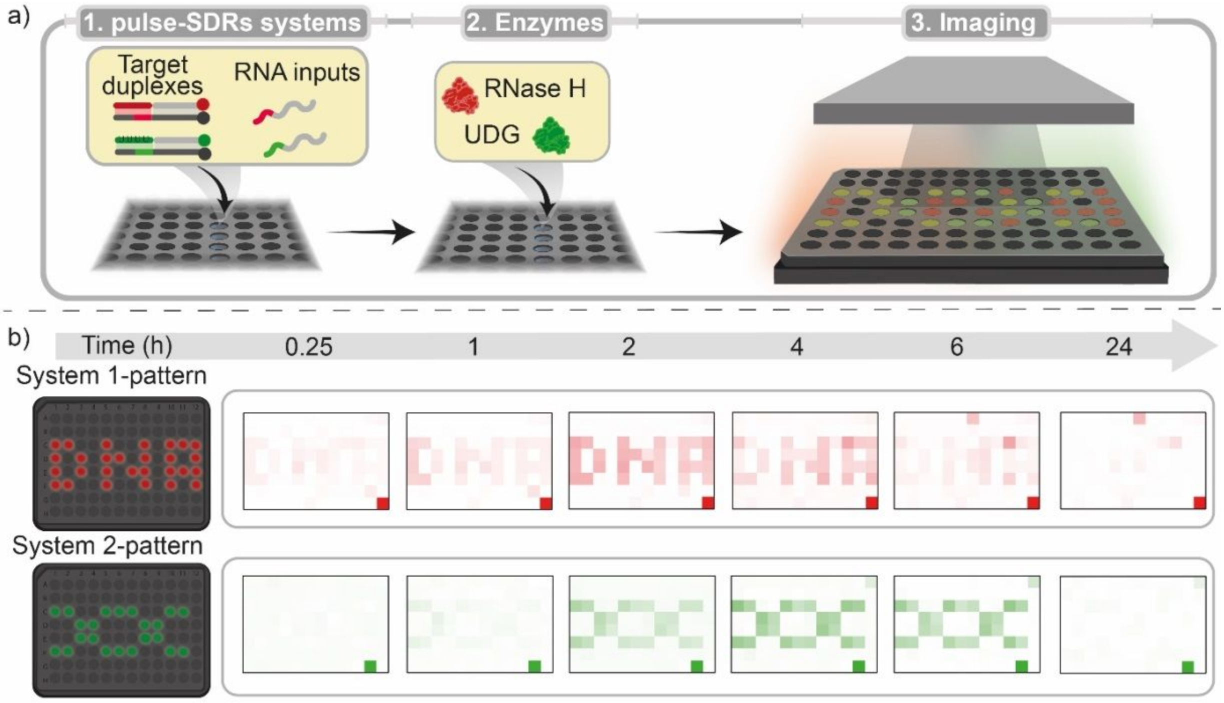

Figure S14. Sequentially-appearing and self-erasing DNA patterns. a) Scheme of the SDR systems used to achieve sequentially-appearing and self-erasing patterns in a 96-well plate. b) Sequential scheme of the 96-well plate resulting in the programmed self-erasing patterns, obtained analyzing the fluorescence time-course experiments at different times. The experiments shown in this figure were performed in Tris HCl 20 mM, $MgCl_2$ 10 mM, EDTA 1 mM, pH 8.0 at T=30°C. Each well contains a fixed concentration of target duplexes (50 nM) and RNA inputs (150 nM) for both systems and different concentrations of the two blocker strands. The two degradation enzymes (RNase H and UDG) were added at the same time in all the wells to achieve the sequential appearing and self-erasing pattern. The wells in the right bottom corner contain a fluorescent reference signal: for system-1 (red) cy3-output strand at 50 nM, for system-2 (green) cy5-output strand at 50 nM.

References

(1) Del Grosso, E.; Irmish, P.; Gentile, S.; Prins, L.J.; Seidel, R.; Ricci, F. Dissipative Control over the Toehold-Mediated DNA Strand Displacement Reaction. *Angew Chem Int Ed*, **2022**, 61, e202201929.

(2) Bucci, J.; Irmisch, P.; Del Grosso, E.; Seidel, R.; Ricci, F. Orthogonal Enzyme-Driven Timers for DNA Strand Displacement Reactions. *J. Am. Chem. Soc.* **2022**, 144, 19791-19798.

(3) Virtanen, P.; Gommers, R.; Oliphant, T.E. *et al.* SciPy 1.0: fundamental algorithms for scientific computing in Python. *Nat Methods*. **2020**, **17**, 261–272.

(4) Hertel, S.; Spinney, R.; Y Xu, S.; Ouldridge, T.; Morris, R.; K Lee, L. The stability and number of nucleating interactions determine DNA hybridization rates in the absence of secondary structure. *Nucleic Acids Res.* **2022**, 50, 7829–7841.

(5) Whitley, K.; Comstock, M.; Chemla, Y. Elasticity of the transition state for oligonucleotide hybridization, *Nucleic Acids Res.* **2017**, 45, 547–555.

(6) Zhang, J., Fang, J., Duan, W.; Wu, L.; Zhang, A.; Dalchau, N.; Yordanov, B.; Petersen, R.; Phillips, A.; Yu Zhang, D. Predicting DNA hybridization kinetics from sequence. *Nature Chem*. **2018**, 10, 91–98.

(7) Panayotou, G.; Brown, T.; Barlow, T.; Pearl, L.; Savva, R. Direct Measurement of the Substrate Preference of Uracil-DNA Glycosylase. *J. Biol. Chem.* **1998**, 273, 45-50.

(8) Bellamy, S.; Baldwin, G. A kinetic analysis of substrate recognition by uracil-DNA glycosylase from herpes simplex virus type. *Nucleic Acids Res.* **2001** , 29, 3857–3863
